# Probiotics mitigate stress and inflammation in malnourished adults via gut microbiota modulation: a randomized controlled trial

**DOI:** 10.3389/fnut.2025.1615607

**Published:** 2025-07-16

**Authors:** Maryam Ahmadi-Khorram, Alireza Hatami, Parastoo Asghari, Ali Jafarzadeh Esfehani, Asma Afshari, Fateme Javdan, Mohsen Nematy

**Affiliations:** ^1^Department of Nutrition, Faculty of Medicine, Mashhad University of Medical Sciences, Mashhad, Iran; ^2^Student Research Committee, Mashhad University of Medical Sciences, Mashhad, Iran; ^3^Metabolic Syndrome Research Center, Mashhad University of Medical Sciences, Mashhad, Iran

**Keywords:** malnutrition, probiotics, perceived stress score, inflammation, stress

## Abstract

**Objective:**

Malnutrition negatively affects mental health by altering neurotransmitter function and increasing stress responses. The gut-brain axis is pivotal in this process, and probiotics may mitigate stress. The current study examined the effects of multi-strain probiotic supplementation on stress levels in underweight individuals using the Perceived Stress Scale (PSS).

**Methods:**

A double-blind, randomized, placebo-controlled trial involved 100 underweight participants were randomized to receive either a probiotic supplement (*Lactobacillus acidophilus*, *L. casei*, *L. rhamnosus*; 3 × 10^9^ CFU) or placebo for 8 weeks. Stress levels, anthropometric measures, and inflammatory markers (ESR, CRP) evaluated at baseline and post-intervention.

**Results:**

Ninety participants (mean age: 26.22 ± 7.42 years) completed the study (probiotic: *n* = 47; placebo: *n* = 43). Baseline age (*p* = 0.051) and gender (*p* = 0.101) showed no significant differences. Post-intervention, the probiotic group exhibited significant weight increases (*p* = 0.005), waist circumference (*p* = 0.038), and hip circumference (*p* = 0.008), and a significant reduction in Perceived Stress Scale (PSS) scores (*p* < 0.001) in comparison to the placebo. Inflammatory markers (ESR, CRP) also decreased significantly in the probiotic group (*p* < 0.001). Within-group analysis revealed improvements in anthropometric measures and inflammatory markers in both groups (*p* < 0.05), but stress reduction was more pronounced in the probiotic group (34% vs. 9.3%, *p* = 0.017). A significant time-group interaction was observed for stress scores (*p* < 0.001).

**Discussion:**

The findings suggest that probiotic supplementation reduces stress levels in underweight individuals, possibly through gut microbiota modulation and inflammation reduction. Further research with larger samples and microbiome analysis is warranted.

**Conclusion:**

In conclusion, administering probiotics to underweight patients positively impacts their mental health and exhibits anti-inflammatory effects.

**Clinical trial registration:**

https://irct.behdasht.gov.ir/trial/69130, identifier IRCT20230310057667N1.

## Graphical abstract

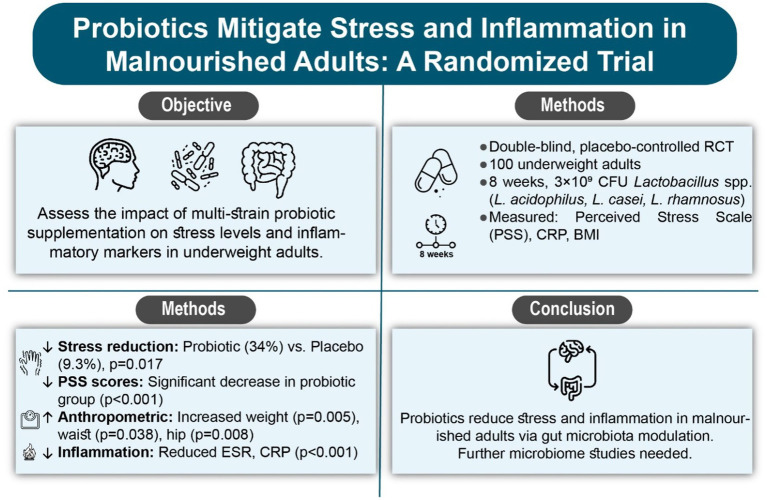

## Introduction

Malnutrition, defined as nutrient deficiencies, excesses, or imbalances, adversely affects body composition, physiological function, and clinical outcomes (1). Undernutrition, specifically underweight conditions, is marked by body weight below healthy standards across age groups (2). In 2022, approximately 183 million women (95% CI: 169–197 million) and 164 million men (95% CI: 148–180 million) were underweight globally, down by 44.9 million women and 47.6 million men since 1990 (3).

Undernutrition disrupts neurotransmitter synthesis (e.g., serotonin, dopamine, GABA), impairing mood, sleep, and stress regulation, and increasing risks of depression and anxiety (4). Chronic malnutrition elevates cortisol, amplifies stress, and, through oxidative stress and inflammation, impairs cognitive functions like memory and attention. This creates a cycle where poor nutrition exacerbates psychological stress, further reducing appetite and worsening health (5, 6).

The gut-brain axis is critical in this interplay. Poor nutrition and gut dysbiosis, mediated by neural, metabolic, and immune pathways, contribute to stress and depression (7). Probiotics, or “psychobiotics,” restore microbiome balance, modulate hormones (e.g., cortisol, serotonin), and reduce pro-inflammatory cytokines (e.g., IFN-γ, TNF-α), alleviating stress and enhancing mental well-being (7).

Probiotics also increase short-chain fatty acid production, which reduces inflammation in conditions like autoimmune disorders and inflammatory bowel disease (8).

While the gut-brain axis provides a promising framework for understanding the interplay between nutrition and mental health, the application of probiotic interventions in underweight individuals is less understood due to limited baseline data on their gut microbiota. While the gut-brain axis and probiotic interventions have been extensively studied in healthy and obese populations, data on gut microbiota alterations in underweight individuals remain limited (9). Undernutrition is associated with reduced microbial diversity, lower SCFA production, and compromised gut barrier integrity, which may uniquely influence the efficacy of probiotics in this population (10). Evidence from healthy or obese cohorts may not fully apply to underweight individuals due to these distinct microbial and physiological profiles. Consequently, this study cautiously interprets the effects of multi-strain probiotic supplementation, recognizing the need for baseline microbiota data specific to underweight individuals to enhance result validity and generalizability.

Recent studies demonstrate mental health benefits: *Lactobacillus casei Shirota* reduced anxiety by 16% and stress by 20% in athletes over 6 weeks (11), while *Lacticaseibacillus rhamnosus HN001* improved happiness and lowered stress in adults after 28 days (12). Synbiotics reduced stress and depression in adults with obesity over 8 weeks (13). Anti-inflammatory effects include lowered hs-CRP in type 2 diabetes and rheumatoid arthritis with *Bacillus coagulans* and *Lactobacillus casei* supplementation (14, 15).

However, the effects of specific multi-strain probiotics (*Lactobacillus acidophilus*, *Lactobacillus casei*, *Lactobacillus rhamnosus*) on psychological stress and inflammation in underweight individuals remain unexplored. This study evaluates the impact of eight-week multi-strain probiotic supplementation on psychological stress, measured via the Perceived Stress Scale, and inflammatory biomarkers in underweight adults, advancing nutritional strategies for mental and physiological health.

## Methods

### Study design and participants

**Figure 1:**
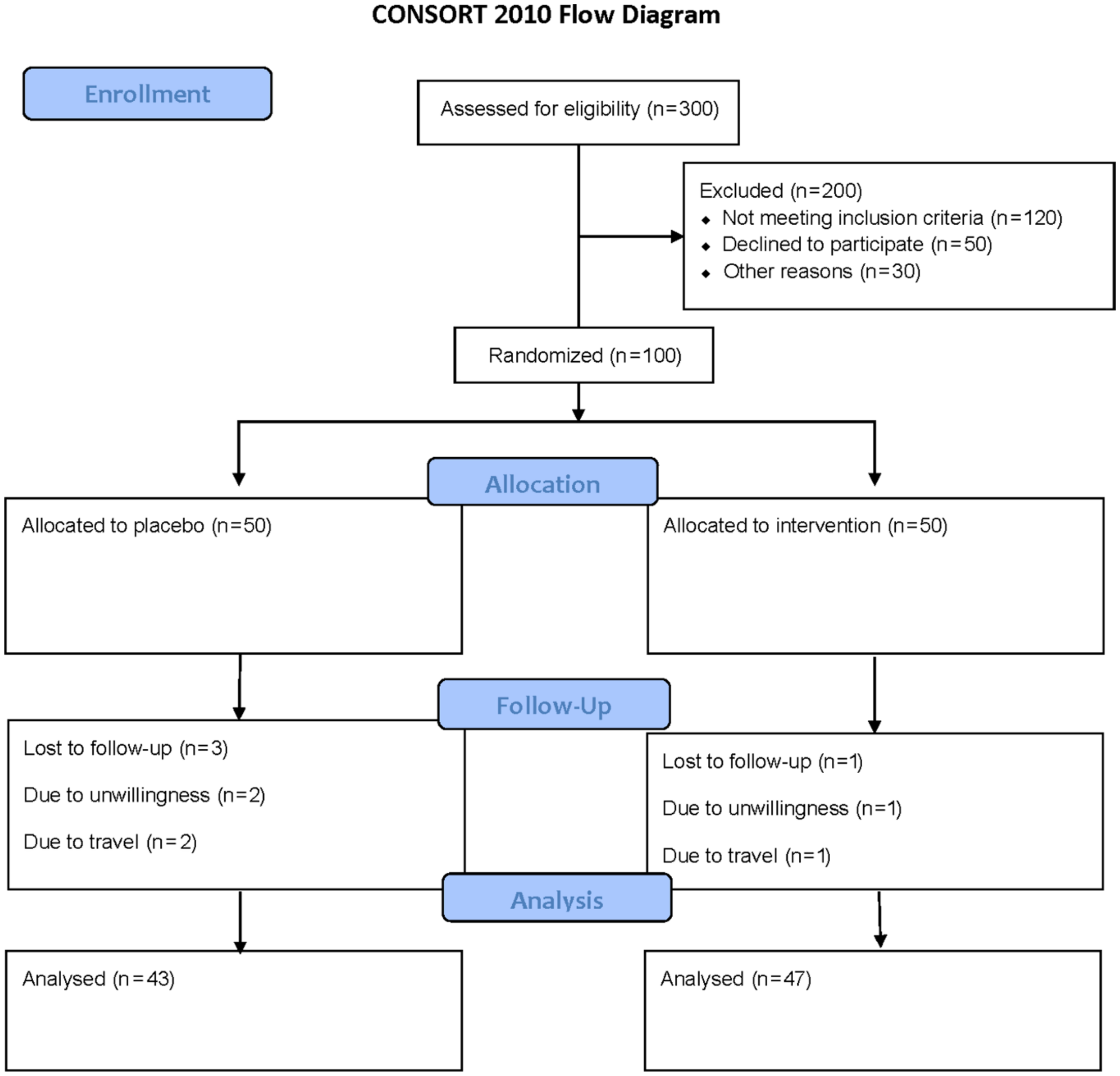
The flowchart of the study. Including patient screening, enrollment, randomization, and follow-up assessments at week 8 for outcome measurements.

This double-blind, randomized, placebo-controlled trial was conducted at the Specialized Nutrition Clinic of Imam Reza Hospital, Mashhad, Iran, between October 1, 2024, and February 28, 2025. The trial was registered with the Iranian Registry of Clinical Trials (IRCT Identifier: IRCT20230310057667N1). Participants were recruited through advertisements and screened for eligibility. Participants were one hundred adults (18–65 years) with undernutrition, defined by BMI <18.5 kg/m^2^ and low FFMI (<17 kg/m^2^ for men, <15 kg/m^2^ for women), assessed to confirm reduced muscle mass. Exclusion criteria included a history of chronic diseases or gastrointestinal disorders, pregnancy, lactation, smoking, and use of antibiotics, probiotics, or foods containing probiotics within 3 months before the study. At baseline, participants completed a detailed questionnaire capturing demographic details (e.g., age, occupation, education), socio-economic status (e.g., household size, housing conditions), medical history, and current medication or supplement use. The sample size was determined based on prior research by Pan et al. (16), which investigated BMI changes following multi-species probiotic supplementation (*Bifidobacterium longum*, *Lactobacillus bulgaricus*, *Streptococcus thermophilus*, 1 × 10^9^ CFU/day) in adults undergoing peritoneal dialysis, a population with nutritional challenges. With an alpha of 0.01, power of 90% (beta = 0.10), and an effect size of 0.4, a minimum of 45 participants per group was required. Allowing for a 10% dropout, 50 participants per group were enrolled, totaling 100 participants (Figure 1).

### Randomization and blinding

Participants were randomized into the probiotic or placebo group using permuted block randomization with a fixed block size of four, ensuring a 1:1 allocation ratio across 25 blocks for 100 participants (50 per group). The randomization sequence was generated by an independent statistician using a web-based platform.^1^ Research assistants enrolled participants after screening, and a study coordinator assigned interventions using numbered, opaque, sealed envelopes to ensure allocation concealment. Both participants and study personnel, including those administering interventions and analyzing data, remained blind to group assignments throughout the study.

### Intervention

Probiotic and placebo capsules, supplied in identical coded containers, contained 3 × 10^9^ CFU of *Lactobacillus acidophilus*, *Lactobacillus casei*, *Lactobacillus rhamnosus*, and maltodextrin filler (probiotic) or maltodextrin alone (placebo), manufactured by ParsiLact Company. Participants were instructed to consume two capsules daily, one after lunch and one after dinner, for 8 weeks. Adherence was monitored via participant self-reported daily logs and capsule counts at week 8, with compliance defined as consuming ≥80% of prescribed capsules. Dietary intake was assessed at baseline and week 8 using 24-h dietary recalls to monitor potential changes. Weekly phone calls and text message reminders were used to reinforce adherence to the probiotic or placebo capsule regimen and to record any reported dietary changes.

### Measurements

Perceived Stress Scale (PSS) questionnaire, blood sampling, and anthropometric indices were measured at the baseline and after 8 weeks.

### Perceived stress scale

The PSS is one of the most commonly used tools for assessing the perception of stress (17, 18). The PSS-10 comprises ten items designed to evaluate the extent to which individuals perceive their life situations as stressful. Each item is scored on a scale from 0 to 4 (0 = never, 4 = very often). Scoring for the PSS-10 was conducted following the guidelines established by Cohen et al. (17) and Cohen (18). Total scores (0–40) categorize stress as none (0), low (0–13), moderate (14–26), or high (27–40).

### Anthropometric indices

Height was measured to 0.5 cm using a stadiometer, weight to 0.1 kg with a digital scale (participants barefoot, lightly clothed), waist circumference at the midpoint between the lowest rib and iliac crest, and hip circumference at the widest point (19).

### Blood sampling

Blood samples (8 mL) were collected at baseline and week 8 from a forearm vein by a trained technician at Navid Laboratory, Mashhad, Iran, between 8:00 and 9:30 AM. Venipuncture used 5-mL EDTA anticoagulant tubes. Samples were centrifuged at 3,000×*g* for 10 min at 4°C within 30 min of collection to separate serum and analyzed immediately. Complete blood count (CBC) was measured using Sysmex KX21, C-reactive protein (CRP) via Roche Cobas 6000 (immunoturbidimetric assay, detection limit 0.1 mg/L, intra-assay CV <5%), and erythrocyte sedimentation rate (ESR) via the Westergren method (ICSH standardized). Derived indices [neutrophil-to-lymphocyte (NLR), platelet-to-lymphocyte (PLR), monocyte-to-lymphocyte (MLR), and neutrophil-lymphocyte-platelet (NLPR) ratios] were calculated. Changes in these markers were analyzed as continuous outcomes using repeated measures ANOVA, with no predefined cut-off points applied.

### Statistical analysis

Data were analyzed using SPSS v24 (IBM Corp, USA). Normality was tested with the Kolmogorov–Smirnov test. Normally distributed variables were reported as mean ± standard deviation (SD), compared within and between groups using paired and independent *t*-tests, respectively. Non-normal data were presented as median (IQR), analyzed with Wilcoxon and Mann–Whitney tests. Categorical variables were compared using chi-square tests. Repeated measures ANOVA was used to evaluate stress scores over time, with baseline variables (age, sex, socio-economic status, BMI) included as covariates to control for confounding effects. All tests were two-sided, with *p* < 0.05 considered significant.

## Results

Ninety participants (mean age: 26.22 ± 7.42 years) completed the study (probiotic: *n* = 47; placebo: *n* = 43). The median age (interquartile range) was 25 (22–32) years for the probiotic group and 23 (20–27) years for the placebo group, with no significant difference (*p* = 0.051, Mann–Whitney test). Gender distribution was similar (male: female ratio was 2.92:1 in the intervention and 1.39:1 in the control group, *p* = 0.101, chi-square test).

Baseline anthropometric measures showed no significant differences (*p* > 0.05). Post-intervention, the probiotic group had significant increases in weight (*p* = 0.005), waist circumference (*p* = 0.038), and hip circumference (*p* = 0.008) compared to placebo. Within the probiotic group, significant improvements occurred in weight (*p* < 0.001), BMI (*p* < 0.001), waist circumference (*p* < 0.001), and hip circumference (*p* < 0.001). The placebo group showed improvements in weight (*p* = 0.003) and BMI (*p* = 0.001). Inflammatory markers differed significantly at baseline for ESR (1 and 2 h, *p* < 0.001 each). Post-intervention, ESR (2 h, *p* < 0.001) and CRP (*p* < 0.001) were significantly lower in the probiotic group. Within-group changes showed significant reductions in ESR (1 h: *p* < 0.001; 2 h: *p* = 0.002) and CRP (*p* = 0.036) in the probiotic group, and ESR (1 and 2 h: plinha <0.001 each) and CRP (*p* = 0.017) in the placebo group (Table 1).

**Table 1:** Comparison of the demographic, anthropometric and laboratory variables between the intervention and control groups.

Variable	Time	Intervention	Control	Between-group *p*
Age (years)		25 (22–32), 95%CI: 24.39–28.55	23 (10–17), 95%CI: 23.60–28.24	0.052^a^
Gender	Male	12 (25.5%)	18 (41.9%)	0.101^c^
	Female	35 (74.5%)	25 (58.1%)	
Height (cm)	Baseline	169.77 ± 9.98, 95%CI: 166.84–172.70	166.60 ± 8.622, 95%CI: 164.68–169.93	0.115^b^
Weight (kg)	Baseline	50.37 ± 8.00, 95%CI: 47.94–52.64	47.73 ± 6.68, 95%CI: 46.34–50.40	0.094^b^
	End of study	52.78 ± 8.41, 95%CI: 50.24–55.17	48.18 ± 6.79, 95%CI: 46.77–50.40	0.005^b^*
Within group *p*		<0.001*	0.003*	
BMI (kg/m^2^)	Baseline	17.6 (16.7–18.5), 95%CI: 16.93–17.81	17.4 (16.4–18.2), 95%CI: 16.88–17.63	0.487^a^
	End of study	18.18 (17.1–19.2), 95%CI: 17.74–18.66	17.5 (16.6–18.5), 95%CI: 17.04–17.94	0.052^a^
Within group *p*		<0.001*	0.001*	
FFMI (kg/m^2^)	Baseline	15.60 ± 1.80, 95%CI: 15.07–16.13	15.20 ± 1.17, 95%CI: 14.86–15.54	0.205^b^
	End of study	15.46 ± 2.32, 95%CI: 14.77–16.14	15.13 ± 1.28, 95%CI: 14.76–15.50	0.406^b^
Within group *p*		0.551	0.340	
Waist circumference (cm)	Baseline	72 (68–75), 95%CI: 69.59–72.41	72 (68–75), 95%CI: 69.36–73.18	0.743^a^
	End of study	74 (70–78), 72.26–75.89	72 (67–75), 95%CI: 69.13–72.75	0.038^a^*
Within group *p*		<0.001*	0.171	
Hip circumference (cm)	Baseline	89 (86–92), 95%CI: 88.00–90.38	87 (85–89), 84.78–88.76	0.052^a^
	End of study	90 (88–94), 95%CI: 85.94–92.75	88 (85–92), 85.85–89.41	0.008^a^*
Within group *p*		<0.001*	0.110	
ESR 1 h	Baseline	5 (3–10), 95%CI: 5.36–7.91	3 (2–5), 95%CI: 3.00–4.92	<0.001^a^*
	End of study	8 (5–15), 95%CI: 3.72–5.25	5 (4–9.25), 95%CI: 4.12–6.30	0.442^a^
Within group *p*		<0.001*	<0.001*	
ESR 2 h	Baseline	3 (3–6), 95%CI: 8.26–11.40	4 (3–7), 95%CI: 5.94–9.73	0.010^a^*
	End of study	6 (4–7), 95%CI: 5.27–7.41	9 (5–13.25), 95%CI: 5.94–9.73	<0.001^a^*
Within group *p*		0.002*	<0.001*	
CRP	Baseline	1 (0.5–3), 95%CI: 1.39–2.28	1.1 (1–4.05), 95%CI: 1.88–3.06	0.198^a^
	End of study	1 (0.3–1.2), 95%CI: 0.85–1.72	4 (1.38–4.8), 95%CI: 3.01–4.76	<0.001^a^*
Within group *p*		0.036*	0.017*	–
NLR	Baseline	1.87 (1.61–2.46), 95%CI: 1.84–2.26	1.75 (1.43–2.63), 95%CI: 1.70–2.11	0.232^a^
	End of study	2.05 (1.32–2.49), 95%CI: 1.85–2.29	1.61 (1.26–2.49), 95%CI: 1.64–2.10	0.180^a^
Within group *p*		0.705	0.440	–
PLR	Baseline	7.83 ± 2.22, 95%CI: 7.17–8.48	7.48 ± 2.22, 95%CI: 6.76–8.05	0.453^b^
	End of study	7.39 (6.25–8.74), 95%CI: 7.12–8.36	7.19 (5.69–8.36), 95%CI: 6.46–8.18	0.371^a^
Within group *p*		0.832	0.340	–
MLR	Baseline	0.15 (0.13–0.20), 95%CI: 0.15–0.18	0.16 (0.11–0.22), 95%CI: 0.14–0.18	0.961^a^
	End of study	0.16 (0.13–0.20), 95%CI: 0.16–0.22	0.14 (0.11–0.19), 95%CI: 0.14–0.18	0.141^a^
Within group *p*		0.262	0.310	–
NLPR	Baseline	0.01 (0.01–0.01), 95%CI: 0.01–0.01	0.01 (0.01–0.01), 95%CI: 0.01–0.01	0.374^a^
	End of study	0.01 (0.01–0.01), 95%CI: 0.01–0.01	0.01 (0.01–0.01), 95%CI: 0.01–0.01	0.175^a^
Within group *p*		0.341	0.378	–

Between-group comparisons are presented in columns and within group comparisons are presented in rows below each variable.

a Median and interquartile range (IQR) were presented, and between-group comparison was performed using the Mann–Whitney test, while within-group comparison was performed using the Wilcoxon test.

b Mean and standard deviation (SD) were presented, and comparison was performed using an independent *t*-test, while within-group comparison was performed using the paired sample *t*-test.

c Frequency and percentage were presented, and comparison was performed using the chi-square test.

*Significant difference.

A comparison of anthropometric and laboratory variables changes between the intervention and control groups is presented in Table 1.

**Figure 2:**
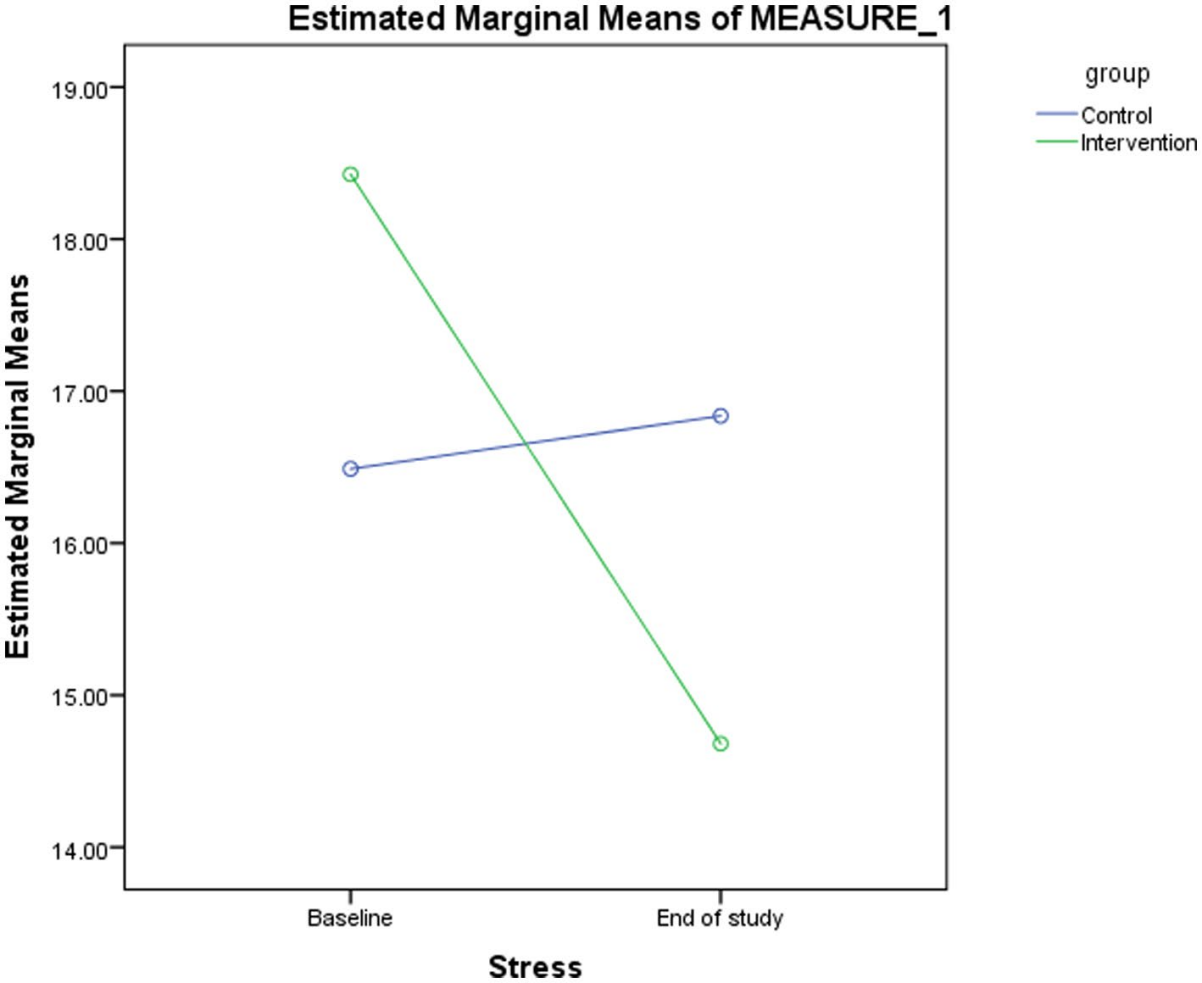
Changes in the estimated marginal means for stress score between baseline and the end of study in the intervention and control groups.

Changes in stress scores between the intervention and control groups are presented and compared in Table 2 and Figure 2. A significant time effect (*p* < 0.001) and time-group interaction (*p* < 0.001) were observed for stress scores in the probiotic group. At the end of the study, stress scores differed significantly between groups (*p* = 0.032). Within the probiotic group, stress scores decreased significantly from baseline to week eight (*p* < 0.001), whereas no significant change was observed in the placebo group (Table 3).

**Table 2:** Comparison of the changes in the anthropometric and laboratory variables between the intervention and control groups.

Variable	Intervention	Control	*p*
Weight (cm)	2.41 ± 1.59	0.45 ± 0.93	<0.001*^b^
BMI (kg/m^2^)	0.8 (0.5–1.1)	0.2 (0–0.47)	<0.001*^a^
Waist circumference (cm)	3 (2–4)	0 (0–1)	<0.001*^a^
Hip circumference (cm)	2 (1–3)	0 (0–1)	<0.001*^a^
ESR 1 h	−1 (−4 to 0)	0.5 (0–2)	<0.001*^a^
ESR 2 h	−1 (−7 to 0)	2 (0–5)	0.001*^a^
CRP	−0.5 (−2 to 0.2)	0.15 (−0.53 to 3.88)	0.001*^a^
NLR	0.03 ± 0.72	−0.04 ± 0.61	0.010*^b^
PLR	0.04 (−1.84 to 1.02)	−0.29 (−1.13 to 0.94)	0.663^a^
MLR	0.01 (−0.03 to 0.51)	−0.01 (−0.05 to 0.03)	0.137^a^
NLPR	0 (0–0)	0 (0–0)	0.180^a^

a Median and interquartile range (IQR) were presented, and between-group comparison was performed using the Mann–Whitney test.

b Mean and standard deviation (SD) were presented, and comparison was performed using an independent *t*-test. *Significant difference.

**Table 3:** Comparison of stress score between the intervention and control groups at baseline and the end of the study.

Variable	Time	Intervention	Control	Time effect *p*	Group effect *p*	Time-group interaction *p*
Stress score	Baseline	18 (16–22)^a^	15 (14–19)	<0.001*	0.905	<0.001*
	End of study	14.68 ± 4.09^ab^	16.84 ± 5.25^b^			

*Significant difference using repeated measures analysis of variance. ^a^*p* < 0.001. ^b^*p* = 0.032.

Comparison of the stress levels between the intervention and control groups at baseline and the end of the study, and changes in stress category over time between groups are presented in Table 4. There was no significant difference in the distribution pattern of stress levels between groups at baseline (*p* = 0.801) or at the end of the study (*p* = 0.108). Of the participants in the control group, the stress level was reduced in four (9.3%), did not change in 37 (86%), and increased in two (4.7%) over the study duration. In contrast, in the intervention group, the stress level was reduced in 16 (34%), did not change in 30 (63.8%), and increased in one (2.2%) participant. A significant difference in stress level changes was observed between groups (*p* = 0.017, chi-square test).

**Table 4:** Comparison of the stress levels between the intervention and control groups at baseline and the end of the study and changes in stress category over time between groups.

Time	Stress level	Intervention frequency (%)	Control frequency (%)	*p*
Baseline	Low	8 (17%)	9 (20.9%)	0.801
	Moderate	37 (78.7%)	33 (76.7%)	
	High	2 (4.3%)	1 (2.3%)	
End of study	Low	21 (44.7%)	12 (36.4%)	0.108
	Moderate	26 (55.3%)	29 (67.4%)	
	High	0 (0%)	2 (4.7%)	

The chi-square test was used for the comparison.

## Discussion

This study is the first to investigate the effects of multi-strain probiotic supplementation (*Lactobacillus acidophilus, Lactobacillus casei, Lactobacillus rhamnosus*) on psychological stress and inflammatory markers in underweight adults. Our findings demonstrate significant improvements in PSS scores and reductions in CRP and ESR levels in the intervention group, highlighting probiotics’ potential in addressing stress and inflammation in this population.

The gut-brain axis, a dynamic network of neural, immune, hormonal, and metabolic pathways, significantly influences mental health (20). Gut microbes regulate brain function by controlling inflammatory markers like interleukin-1, which can trigger cortisol release through the hypothalamic–pituitary–adrenal axis (21). Additionally, short-chain fatty acids (SCFAs) produced by gut microbiota contribute to mental health by modulating the immune system and neurotransmitter production. Probiotics enhance gut microbiome composition, reinforcing the intestinal barrier and producing antimicrobial substances that support mental well-being (22). To further elucidate the mechanisms underlying these effects, the potential direct and indirect actions of the probiotic strains used in this study warrant exploration. The observed improvements in PSS scores and reductions in CRP and ESR levels may result from both direct and indirect effects of the multi-strain probiotic supplementation. Directly, Lactobacillus strains may produce bioactive metabolites, such as short-chain fatty acids (SCFAs), which modulate immune responses and neurotransmitter synthesis, enhancing mental well-being (20–22). These strains may also directly interact with the hypothalamic–pituitary–adrenal (HPA) axis, potentially reducing cortisol levels by downregulating pro-inflammatory cytokines (e.g., IL-1, TNF-α) that stimulate cortisol release (21). Indirectly, probiotics may alter gut microbiota composition, strengthening the intestinal barrier and reducing systemic inflammation, although some studies suggest probiotic supplementation does not always significantly change microbiota composition (23). The absence of microbiome analysis in this study limits our ability to confirm these mechanisms. Future research should include microbial profiling to elucidate whether the observed effects are primarily driven by direct probiotic actions or microbiota-mediated changes.

SCFAs, such as acetate, propionate, and butyrate, produced by Lactobacillus strains, likely contribute to the observed reductions in PSS scores and inflammatory markers by modulating the gut-brain-immune axis. SCFAs enhance GABAergic activity by upregulating GABA receptor expression in the brain, potentially reducing stress and improving emotional regulation (24). Additionally, SCFAs inhibit pro-inflammatory cytokines (e.g., IL-6, TNF-α) by suppressing NF-κB signaling, which may explain the reductions in CRP and ESR levels (25). Although IL-6 and TNF-α were not measured in this study, their involvement in SCFA-mediated immune modulation suggests a mechanistic pathway for future investigation. These effects underscore the role of SCFAs in linking gut microbiota to brain function and systemic inflammation.

Research into natural alternatives for cognitive and mental health improvement has expanded, leading to the concept of ‘psychobiotics,’ probiotics that confer mental health benefits (26). Probiotics influence the gut-brain axis and provide a natural approach to managing stress and enhancing mental health outcomes (27). Several clinical trials have examined probiotics’ effects on psychological health, with varying results depending on the population and probiotic strain used (12).

Studies on *Lactobacillus rhamnosus* have reported both positive and neutral results. For example, supplementation with *Lactobacillus rhamnosus* improved depressive symptoms and quality of life in post-myocardial infarction patients (28), and reduced postnatal depression and anxiety in pregnant women (29). However, no significant benefits were observed in university students or healthcare workers during the COVID-19 pandemic (30, 31). These discrepancies suggest that probiotic efficacy may depend on population characteristics and external factors. *Lactobacillus casei* supplementation has been shown to improve sleep quality and reduce stress-related symptoms in medical students during exams and athletes under competitive pressure (11, 32). Furthermore, synbiotic supplementation combining *Lactobacillus acidophilus* with other strains has demonstrated reductions in stress, anxiety, and depression in individuals with various conditions (13, 33, 34).

Regarding inflammatory markers, ESR and CRP levels decreased in the intervention group, but the between-group differences were not statistically significant, possibly due to the small sample size or low baseline inflammation levels. The use of more sensitive biomarkers, such as IL-6 or TNF-α, may better capture subtle inflammatory changes in future studies (35). This aligns with previous studies that reported reductions in hs-CRP levels following probiotic supplementation in patients with rheumatoid arthritis, coronary artery disease, and type-2 diabetes (14, 15, 36). The reason for the no significant difference observed in NLR, PLR, MLR and NLPR was hypothesized to be due to the changes in these parameters being within the normal range. Limitations include the absence of microbiome analysis to clarify mechanisms and a modest sample size that limits generalizability. Future research should involve larger samples and microbial profiling to optimize probiotics interventions. This study did not evaluate gut permeability, absorption efficacy, psychological effects and other possible factors that might affect the outcomes. Therefore, it I suggested that further studies evaluate these factors and the mechanism of the observed effects.

## Conclusion

In conclusion, probiotic supplementation in underweight patients benefits mental health and reduces inflammation in underweight adults, offering a complementary approach to stress management. Further studies are needed to validate these findings and explore probiotics as a primary stress intervention.

## Data Availability

Data described in the manuscript, will be made available upon written request to the corresponding author and approval by the Vice Chancellor of Research and technology of the Mashhad University of Medical Sciences.

## Footnotes

^1^ https://www.sealedenvelope.com
